# Holocellulose from a Winemaking By-Product to Develop a Biopolymeric System for Bacterial Immobilization: Adsorption of Ochratoxin A in Wine Model Solutions (Box–Behnken Design)

**DOI:** 10.3390/toxins17010026

**Published:** 2025-01-06

**Authors:** Verónica Carrasco-Sánchez, V. Felipe Laurie, Marcelo Muñoz-Vera, Ricardo Ignacio Castro

**Affiliations:** 1 Departamento de Microbiología, Facultad de Ciencias de la Salud, Universidad de Talca, Talca 3460000, Chile; 2 Centro de Nanomedicina, Diagnóstico & Desarrollo de Fármacos, Universidad de Talca, Talca 3460000, Chile; 3 Facultad de Ciencias Agrarias, Universidad de Talca, Talca 3460000, Chile; flaurie@utalca.cl; 4 Construction Multidisciplinary Research Group, Facultad de Arquitectura, Construcción y Medio Ambiente, Universidad Autónoma de Chile, Talca 3460000, Chile; marcelomunoz132@gmail.com; 5 Multidisciplinary Agroindustry Research Laboratory, Carrera de Ingeniería en Construcción, Instituto de Ciencias Químicas Aplicadas, Universidad Autónoma de Chile, Talca 3460000, Chile

**Keywords:** holocellulose, stalk grape, bacterial immobilization, alginate, chitosan, ochratoxin A

## Abstract

Significant agro-industrial waste is produced during the winemaking process, including grape stalks, which are a rich source of the valuable biopolymer holocellulose that can be utilized for biotechnological processes. The purpose of this study was to delignify grape stalks in order to extract holocellulose. Then Lactobacillus plantarum (LP) was immobilized in the interstitial spaces of holocellulose and then coated with natural polymers (chitosan, Ch; and alginate, Al) to create the Holo-LP/Ch/Al complex. A physicochemical analysis of the system revealed strong bacterial immobilization and stability. The efficiency of the complex in adsorbing ochratoxin A (OTA) from wine model solutions was assessed using a Box–Behnken design under various pH, time, and concentration conditions. The results showed that at pH 3.0, 75.39 min, and a complex concentration of 43.82 mg mL^−1^, the best OTA removal (53.68%) took place. Because of its physicochemical interactions, the complex showed improved OTA adsorption in acidic environments. This study demonstrates the potential of biopolymeric systems based on holocellulose for reducing mycotoxin contamination in beverages and stabilizing bacterial cells. These results offer a viable way to increase food safety and value winemaking by-products.

## 1. Introduction

The wine industry, a key player in biotechnological activity, produces a significant number of by-products. For every 100 L of wine, approximately 30 kg of by-products are generated, including 13.2–17 kg of skins and 4 kg of stalks [1]. While these grape skins and stalks are not hazardous waste, their high organic matter content and seasonal production can lead to potential pollution problems [2]. More importantly, the current disposal methods are not economically advantageous, leading to direct costs for wineries or indirect costs for the community. This economic burden underscores the urgent need for viable alternatives.

Grape stalks, the skeletal remains of grape bunches, are primarily composed of holocellulose (15 at 40% cellulose, 12 at 30% hemicelluloses), lignin 15–30%, and extractives such as water, sugars, acids, and phenolic compounds [2,3,4]. Traditionally, this residue has been used solely as a fertilizer after composting. However, recent research has explored innovative uses for this waste, such as the extraction of biopolymers like cellulose and hemicellulose, highlighting the potential for waste valorization in the wine industry [5]. One way to use these polymers is to immobilize microorganisms, other cells, or enzymes for biotechnological applications. This can help make food and drinks safer to consume. For example, these materials can be used to remove harmful substances like mycotoxins from beverages like beer, wine, and juice [6,7,8,9].

Mycotoxins are small molecules produced by the secondary metabolism of certain types of fungi, such as Fusarium, Aspergillus, and Penicillium [10,11]. These fungi can contaminate a wide range of crops [12,13], with mycotoxins present in around 60% to 80% of crops [14]. Some mycotoxins have been linked to health problems, ranging from acute poisoning to chronic conditions like cancer, immune system disorders, kidney and liver damage, and neurological issues [15]. These issues can also lead to significant economic losses in affected industries.

The main mycotoxin found in wines and other beverages is ochratoxin A (OTA) [6,7,8,9], which is primarily produced by *Aspergillus* and *Penicillium* species [16]. OTA is harmful to the kidneys, liver, immune system, and nervous system, and it is also known to be teratogenic and potentially carcinogenic. The International Agency for Research on Cancer (IARC) has classified OTA as group 2B, meaning it is possibly carcinogenic to humans [17]. The European Food Safety Authority (EFSA) [18] has established the maximum allowable levels of this toxin at 2 µg/kg for beverages, including wines and grape juices, that are intended for direct human consumption [19].

Chemical and physical methods have been explored to eliminate or reduce mycotoxin contamination from beverages [20]. However, these methods do not guarantee that drinks are entirely free of mycotoxins [12]. Currently, biological methods are gaining importance. For example, the use of lactic acid bacteria (LAB) with mycotoxin adsorption properties has emerged as a promising alternative for removing mycotoxins from liquid substances [21,22]. An important aspect of LABs is that they have been included in the Qualified Presumption of Safety (QPS) list for authorized use in the food and feed chain within the European Union. Furthermore, in the US, they are classified as GRAS, which stands for “generally regarded as safe” [18,23].

Based on laboratory studies, certain species like *Lactobacillus rhamnosus*, *L. acidophilus*, *L. plantarum*, *L. lactis*, *Streptococcus thermophilus*, and *Bifidobacterium bifidum* have been found to effectively retain mycotoxins from liquid matrices. Each species has different effects on various mycotoxins. *L. rhamnosus* and *L. plantarum* are particularly versatile, being able to efficiently remove several mycotoxins at the same time [24,25]. Research has shown that these microorganisms can adsorb approximately 20% to 90% of mycotoxins in different liquid food matrices [25,26,27,28,29,30].

The ability of microbial cells to remove mycotoxins is generally good in laboratory conditions or model matrices (such as water or buffers). However, free microbial cells can face significant challenges, including survival, proliferation, mechanical disturbances, low adaptation, and competition from indigenous microorganisms in natural environments [31,32]. To address these issues, immobilization strategies have emerged as a promising alternative for using lactic acid bacteria in the removal of mycotoxins from beverages [33].

Polymer configurations have been developed for immobilizing bacteria, such as entrapping them in a polymeric matrix [34] or attaching them to a solid support [35]. The support materials need to be insoluble, non-toxic, non-polluting, lightweight, flexible in shape, mechanically and chemically stable, easy to immobilize bacteria on, capable of retaining high biomass, and preferably low-cost.

While inorganic polymers like zeolite, clay, porous glass, activated charcoal, and ceramics are commonly used for bacterial immobilization [36], there is a growing interest in natural polymers. Natural polymers such as alginate, carrageenan, agar, agarose, cellulose, lignin, and chitosan are gaining popularity due to their ease of handling, non-toxic nature, safety for human use and the environment, and their ability to trap microorganisms [37,38] Numerous studies have explored the use of cellulose membranes to immobilize microorganisms, indicating their potential for various environmental applications [39]. However, the weak adhesion between bacteria and nanofibrous membranes poses a significant challenge for real-world industrial use. To address this issue, the proposal is to immobilize bacteria onto the holocellulose structure in the interstitial space created by removing lignin from grape stalks and then forming a coating with natural polymers for enhanced stability and cell protection. Natural polymers such as alginate and chitosan are suggested alternatives due to their ability to form anionic/cationic complexes, creating crosslinked systems that can provide structural support [40,41,42].

**Figure 1:**
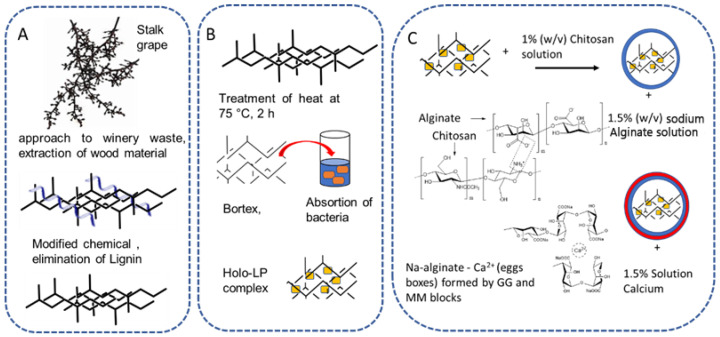
Development of a biopolymeric system for bacterial immobilization. (**A**) Lignin elimination from stalk grape; (**B**) Holo-LP complex formation; (**C**) Holo-LP/Ch/Al Complex Formation.

This work aimed to obtain holocellulose from vinifera grape waste and use it, along with alginate and chitosan, to create polymeric support for immobilizing lactic acid bacteria to be used in the removal of mycotoxins from a wine model solution (Figure 1).

## 2. Results and Discussion

### 2.1. Thermogravimetric Analysis (TGA) and Derivate Thermogravimetry (DTG) of Holocellulose

**Figure 2:**
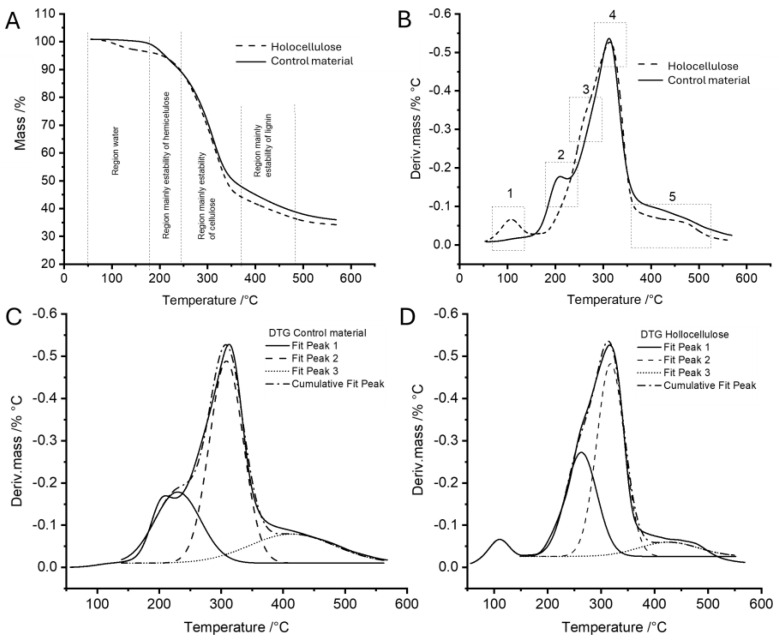
Analysis of control and holocellulose: (**A**) TG curve; (**B**) DTG curves; (**C**) deconvolution curves of control; (**D**) deconvolution curves of holocellulose.

The TGA results in Figure 2 show that hemicellulose and lignin decompose, leading to increased water absorption (Figure 2A,B). This is mainly due to mass loss and the creation of spaces that allow for better absorption. On the other hand, the untreated sample demonstrates greater thermal stability in the TGA and DTG thermograms, primarily because of the strong network formed among the polymer components. The decrease in thermal stability of the treated material is attributed to the degradation of lignin and some of the hemicellulose. This is evident in the DTG peaks, which show differences between treated and untreated samples at 210 and 260 °C, indicating hemicellulose decomposition. The remaining portions between 310 and 422 °C may result from cellulose and lignin degradation. These findings align with the literature cited by Castro and Morales-Quintana (2019) [40]. As the temperature reaches 450 °C, it becomes clear that most of the mass losses result from lignin degradation, along with structural changes and ash formation, as indicated by Hu et al. (2014) [43].

The impact of lignin extraction processes on the thermal degradation behavior of wood components highlights the importance of lignin in determining the overall stability of the samples. After removing the lignin, the samples showed similar thermal stability, indicating a neutralizing effect on the degradation process. However, the subtle differences in specific features after extraction suggest that delignification has a broader impact. Regarding specific temperature features, the shift of signals related to hemicellulose in Figure 2C suggests a change towards higher temperatures. This shift contributes to the degradation of weaker structures, indicating a complex relationship between lignin extraction and the thermal behavior of hemicellulose. Similarly, in the 2D graph, the peak displacement from the cellulose region towards higher temperatures can be attributed to the degradation of amorphous cellulose, while the stability of crystalline cellulose indicates that amorphous regions of cellulose are more accessible and susceptible to degradation [44].

The deconvolution of thermograms has provided additional insights, revealing a decrease in lignin content from 19.6% to 8.8% (refer to Figure 2C,D and Supplementary Materials, Tables S1 and S2). This decrease confirms the successful isolation of holocellulose, further supported by the thermal profiles obtained. These results collectively indicate that the processes for extracting lignin not only affect overall thermal stability but also cause specific changes in the degradation behavior of individual wood components. This contributes to a more detailed understanding of the thermal dynamics in the resulting holocellulose.

### 2.2. FTIR Analysis of Delignification

**Figure 3:**
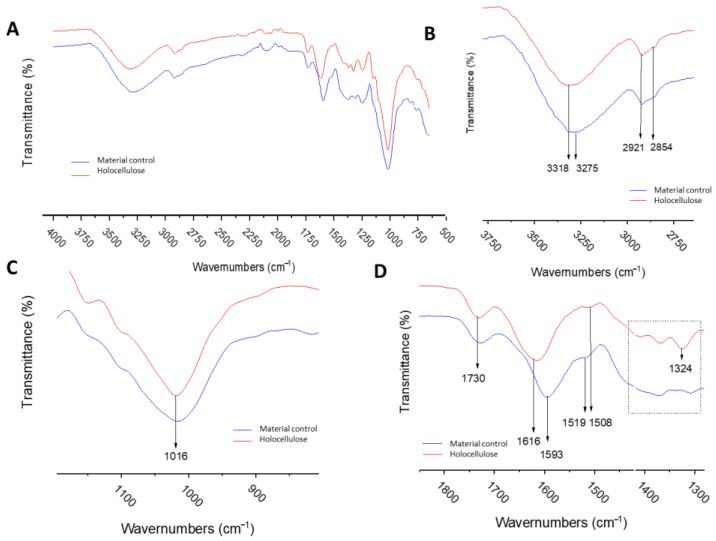
(**A**) FTIR spectra, holocellulose (red line), and control (blue line); (**B**) range between 3800 at 2700 cm^−1^; (**C**) range between 1200 at 800 cm^−1^; (**D**) range between 1800 at 1300 cm^−1^.

The FTIR analysis of both treated and untreated grape residue samples is shown in Figure 3. The results indicate significant changes in the signals at 3318 cm^−1^, which are related to the presence of hydroxyl groups in both samples. However, in Figure 3B, there is a shift in the signal to 3275 cm^−1^ with increased intensity, indicating the removal of hydroxyl groups from lignin and partial loss of hemicellulose (the signal of O_6_H_6_O_3_ intermolecular in cellulose Iβ stretching) [45].

Additionally, Figure 3C displays broad signals ranging from 1050 to 950 cm^−1^, with a peak at 1016 cm^−1^, primarily attributed to C−O stretching from C(3)−O(3)H in cellulose I and C−O and C−C stretching ring in cellulose and hemicelluloses [46], confirming the preservation of cellulose and part of hemicellulose post-treatment. Moreover, Figure 3D also exhibits signals confirming the presence of these structures before and after treatment, particularly at 1730 cm^−1^, indicating C-O stretching in unconjugated ketone, carbonyl, and aliphatic groups (xylan) [47]. However, the signal at 1593 cm^−1^ disappears in the treated samples, signifying C=C stretching of aromatic compounds, a peak typically attributable to lignin (C=C stretching vibration). This alteration primarily results from the decomposition of the lignin structure, further supported by the disappearance of signals at 1519 cm^−1^, associated with aromatic skeletal stretching (lignin) [48], due to control delignification. The characteristic bands in FTIR spectra were observed in Table 1.

### 2.3. Color Analysis After Delignification Process

The process of obtaining holocellulose involves removing lignin from wood, which results in changes in color and the loss of lignin. This is because lignin is responsible for the natural yellow-brown color of wood. However, the removal of lignin also increases the ability of the timber to form microporosity, making it more suitable to produce materials that can be used as an absorption system.

The outcome shows an increase in the L* value, which indicates a clearer color. Meanwhile, the a* and b* values decreased, indicating a shift towards red and yellow colors, respectively. Finally, the changes in color were expressed as ΔE* according to Equation (1) (ΔE* = 18.4), and as a result, the samples exhibited “distinct color changes” (see Table S1 in Supplementary Materials) from three replicates.

### 2.4. Morphological Analysis by Scanning Electronic Microscopy (SEM)

**Figure 4:**
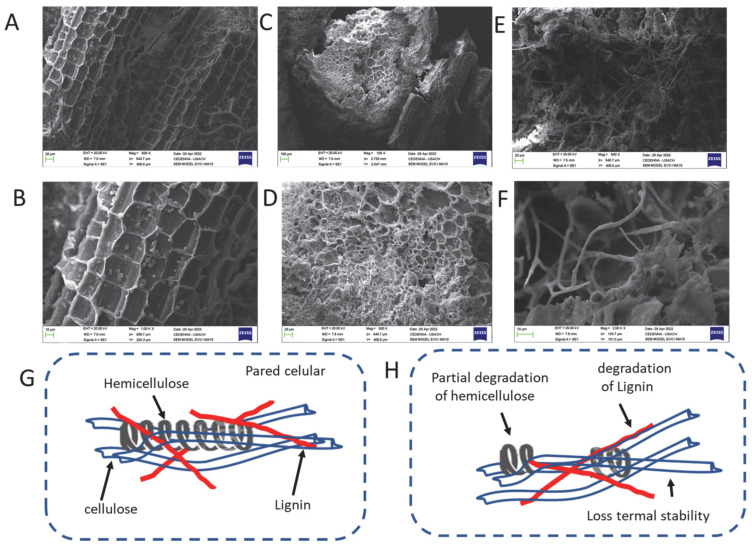
Scanning electronic microscopy (SEM); (**A**,**B**) waste from grape stalk of *Vitis vinifera* L. (control); (**C**,**D**) changes in waste due to mechanical; (**E**,**F**) Holocellulose; (**G**,**H**) representation of the formation of holocellulose.

Several structural differences were observed between the treated and untreated grape stalks. When the lignin and holocellulose were separated during the extraction and processing, the stalks showed morphological changes. As seen in Figure 4A,B, there is a multilayered, densely compacted polymeric structure. On the other hand, Figure 4C,D depict morphological changes that resulted from mechanical wear during the grinding process.

Figure 4E,F illustrate that distinct voids appeared after treatment with sodium chlorite, causing the microscopic particles to almost entirely disappear and generate a network of crosslinked polymers with spaces or pores due to the absence of lignin. This resulted in a low-density structure, as lignin typically determines the microporosity of wood-derived structures. In contrast, cellulosic degradation during the treatment with sodium chlorite NaClO_2_ produces volatile compounds [57].

### 2.5. Syntheses and Characterization of Holo-LP/Ch/Al Complex

#### 2.5.1. Holo-LP/Ch Complex Formation

Bacteria are inserted onto dehydrated bleached cellulosic material through a type of diffusion. The bacteria form a complex with holocellulose, which is a similar process to osmosis. This process is driven by the high concentration of bacteria in water, moving from an area of high concentration to an area of low concentration without requiring energy. The dehydrated holocellulose absorbs the bacteria into the spaces of the fibers, ensuring that the bacterial mix can fully penetrate the material.

To form the Holo-LP/Ch complex, we used chitosan at a concentration of 1.0% *w*/*w* while maintaining a neutral pH. This helped to generate a stable complex with the holocellulose–bacteria structures, which were covered with a layer of chitosan. This stability is because cellulose and chitosan share similar structures, with the only difference being that the C_2_-OH of cellulose is substituted by the C_2_-NH_2_ of chitosan. This results in reinforced molecular interactions between their chains [58]. These interactions enhance the chemical stability of chitosan, creating a more stable complex [59].

In this study, we aimed to create a complex that can gel in the presence of calcium ions [60]. The interaction of hydroxyl ions of cellulose or hemicellulose with amino groups of chitosan is necessary to form ionic bridges [61]. These structures are needed to contain *Lactobacillus plantarum* in their interiors (interstitial spaces). The holocellulose–bacteria–chitosan complex is introduced dropwise into the alginate solution so that the remaining amino groups form a third layer of interaction with alginate.

#### 2.5.2. Holo-LP/Ch/Al Complex Formation

The chitosan–cellulose–bacteria was coated with an alginate layer, which was crosslinked by adding the solution to calcium chloride. This captured the Ca^2+^ ions, forming “egg boxes” by interacting with GG and MM blocks. Previous studies have shown that the Na-alginate polymer can bind with Ca^2+^ ions in aqueous solution [62]. The high surface area and numerous functional groups on the surface not only increase the effectiveness of chitosan’s adsorption but also allow for electrostatic interactions between chitosan and alginate [63]. Additionally, they can act as a physical crosslinker through hydrogen bonding interactions with the matrix’s functional groups.

Alginate and chitosan can form polyelectrolyte complexes through ionic interactions, which makes them useful for various applications. It is believed that the protonated amino groups on the chitosan and alginate equivalents will ionically interact to create a three-dimensional matrix called a physically crosslinked hydrogel [64]. Because of their opposite charges, if alginate and chitosan solutions are mixed directly, they would quickly congeal or gel, making it impossible to produce the corresponding alginate/chitosan fibers from the combined solutions of the polymers. Using conventional microscopy, we assess the presence of lactic acid bacteria immobilized in the formed polymer by staining the complexes (see Figure S1 in Supplementary Materials).

#### 2.5.3. Analysis Thermogravimetric of Holo-LP/Ch/Al Complex

**Figure 5:**
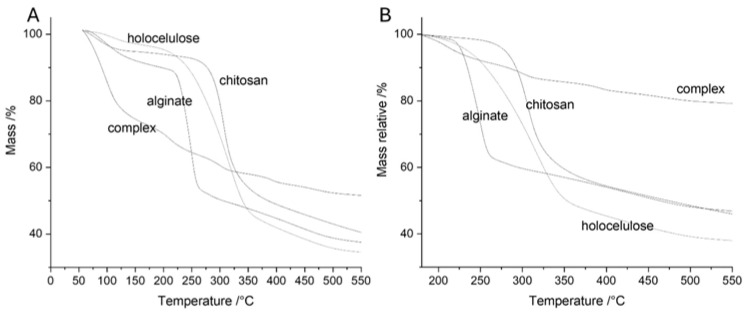
TGA of *Holo-LP/Ch/Al* complex and controls: (**A**) TG curve; (**B**) DTG curves relative loss mass.

The thermogravimetric analysis examined the thermal stability of all the polymers used in this study. The findings show that chitosan has higher thermal stability than alginate (see Figure 5). Furthermore, chitosan demonstrated greater thermal stability than holocellulose, possibly due to the linear structure of chitosan, which allows for increased interactions between its chains, resulting in greater stability.

On the other hand, the reduced stability of holocellulose is due to the breaking of important bonds during the delignification process [65]. This results in spaces between the fibers and a decrease in density due to structural expansion (see Figure 4). Additionally, the concentration of adsorbed or absorbed water for the three dehydrated structures analyzed does not exceed 10%. In contrast, the formed complex reaches 27%, indicating a significant difference (Table S3 in the Supplementary Materials). This water contributes to the separation of the structure and the formation of new hydrogen bonds throughout the structure, facilitated by the hydroxyl groups present. Moreover, the complex shows ionic interactions between the layers, including interactions between the carboxyl group (-COOH) of holocellulose, which is mainly present in hemicellulose, and the amino groups (-NH_2_) of chitosan [66].

The primary structure surrounding the Holo-LP complex is made up of an interpenetrating polymer network of alginate-chitosan chains with amide bonds. Cationic amino groups of chitosan on the periphery of this structure interact with the carboxylic groups of alginates, leading to the formation of a second layer due to electrostatic interactions [67]. This second layer is further stabilized by bivalent calcium ions interacting with carboxyl groups of alginates. This creates a stable gel-like structure known as an “egg box” array [62]. Additionally, coordination between the calcium, guluronic, and mannuronic acid groups contributes to the formation of a highly stable complex capable of encapsulating lactic acid bacteria within a shell-like system.

#### 2.5.4. FTIR Analysis of Holo-LP/Ch/Al Complex

**Figure 6:**
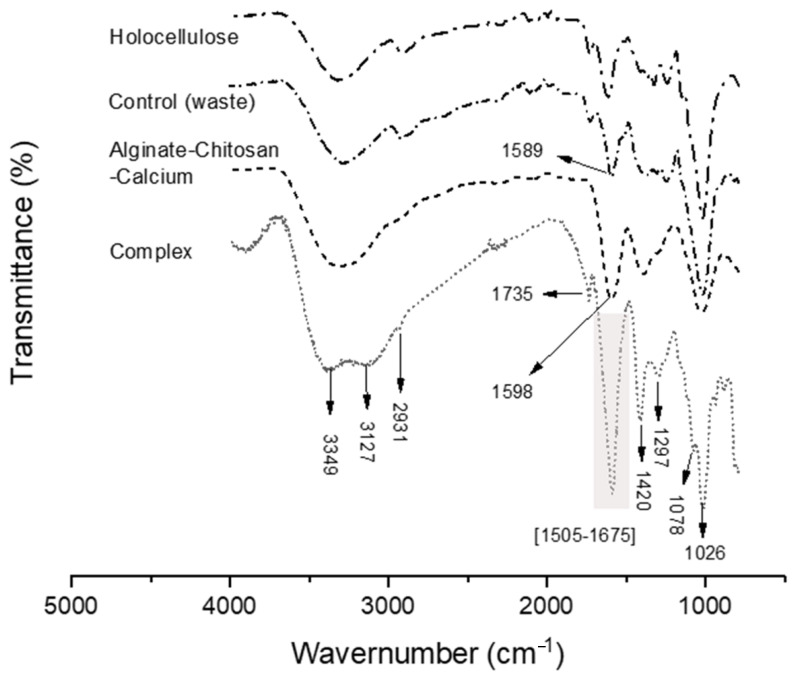
FT-IR spectra of different samples.

The FTIR spectrum of the complex synthesized from holocellulose, alginate, chitosan, lactic acid bacteria, and CaCl_2_ is illustrated in Figure 6 and described in Table 2. The signals reveal a prominent broad band ascribed to the OH stretch at 3349 cm^−1^, primarily originating from alginate. This band overlaps with the N–H stretching at 3127 cm^−1^ of chitosan [68,69]. Furthermore, notable bands at 2931 and 1420 cm^−1^ are observed, corresponding to the stretching and bending frequencies of the –CH_2_ groups present in both alginate and chitosan; the peaks at 1643 and 1078 cm^−1^ are attributable to the bending and stretching vibrations of –NH_2_ groups (chitosan) and –COO– groups (alginate), respectively [70], confirming the presence of both polymers in the complex (chitosan–alginate); however, a signal that demonstrates the presence of holocellulose is the signal indicative of C-O stretching in unconjugated ketone, carbonyl, and aliphatic groups (xylan) are observed within the spectral range of 1730–1726 cm^−1^ [47]. Lastly, the spectrum exhibits several overlapping signals, representing a combination of various components within the complex. These include the N-H bending from amine and amide II at 1574 cm^−1^ [71], as well as the antisymmetric COO- vibration (alginate) at 1596 cm^−1^ [68].

### 2.6. OTA Removal by Holo-LP/Ch/Al Complex

#### 2.6.1. Response Surface Analysis Models

The study used a Box–Behnken design (BBD) to optimize the process parameters for removing OTA using a polymeric Holo-LP/Ch/Al complex. The parameters studied were pH, time, and concentration. This model was used to improve experimental techniques for evaluating several variables at once and their interactions. This statistical method guarantees the parameter analysis (such as pH, concentration, and exposure duration). By reducing the number of experimental runs required and increasing the quality of the results, the Box–Behnken model assists us in determining the ideal values for these variables.

The results of OTA removal from different experimental runs are summarized in Table S4 in the Supplementary Materials.

**Figure 7:**
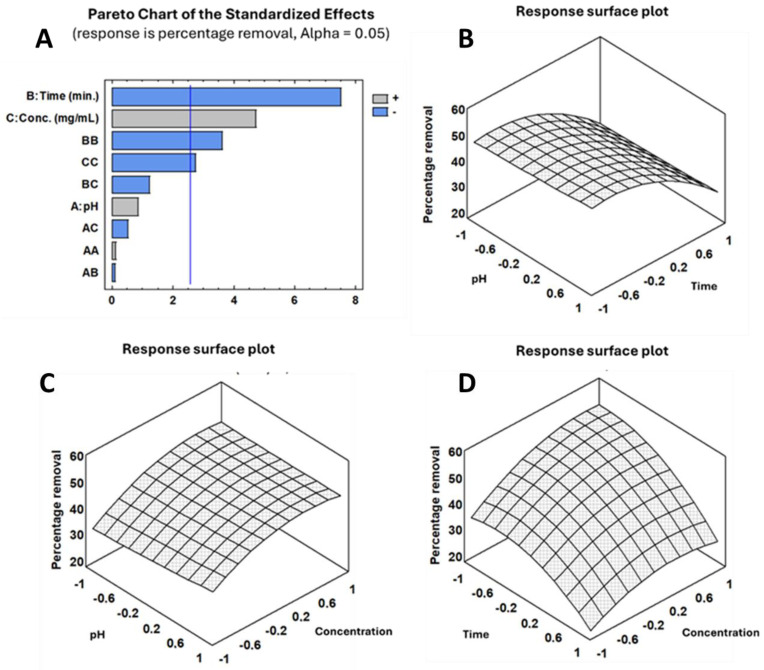
(**A**) Pareto chart standardized effects; (**B**–**D**) three-dimensional response surface plot for the analytical response vs. the effect of A (pH), B (time; min.), and C (Concentrations; mg mL^−1^).

The removal efficiency was analyzed using Pareto charts, which displayed significant variation with changes in pH (refer to Figure 7). For example, at a lower pH of 3.0, the removal efficiency reached up to 49.83% (Run 9). In contrast, at a higher pH of 3.5 and low concentration (10 mg mL^−1^), the removal efficiency was lower, at 23.549% for Run 7 and 34.9% for Run 7 at pH 4.0. It indicates that the polymeric complex has a stronger attraction for OTA under more acidic conditions, thus enhancing the adsorption process. This behavior is likely due to the proximity to the p*K*_a_ of the acidic group in OTA. At more basic pH levels, the acidic group would be negatively charged, leading to steric hindrance that prevents it from being absorbed by the complex [74].

The efficiency of OTA removal was influenced by the duration of exposure to the polymeric complex. Longer exposure times (240 min) generally resulted in higher removal efficiencies compared to shorter times (60 min). For instance, at pH 3.0 and a specific concentration, the removal efficiency was 48.9% (Run 2) at 240 min, compared to (Run 7) at 150 min. Regarding the effect of concentration, the removal efficiency tended to stabilize at the highest concentration, possibly due to the saturation of the polymeric adsorption sites.

It is an established fact that *L. plantarum* influences the adsorption capacity of OTA by the complex. Previous studies have shown that when *L. plantarum* is immobilized in a polymeric system, it can enhance the ability to remove OTA from wine by about 10% [41]. Furthermore, the variability in *Lactobacillus* strains has shown promising results, with different strains capable of removing 8 to 28% of the OTA from wine, depending on the strain [75]. Importantly, these findings have practical implications, as the successful application of LP in real samples has shown that it can facilitate OTA removal without significantly affecting phenol removal, providing reassurance about its effectiveness in real scenarios.

It is important to note that this study is a proof of concept in the laboratory; the use of a high dose of ochratoxin A, while not representative of a truly contaminated wine, has been used in previous studies to establish and optimize parameters on a laboratory scale.

Let us examine the global situation concerning the contamination of wines by ochratoxin A. Reports indicate that Italian wines have an OTA concentration of 2 ng mL^−1^. In wines from the United States, the concentrations vary between 0.3 and 8.6 ng mL^−1^, while in Paraguay, concentrations of 2.4 ng mL^−1^ have been documented [76].

It is established that although these concentrations are low, they are at the limit or above the maximum limit permitted by the European Union; therefore, the fact that Holo-LP/Ch/Al complex can effectively reduce high concentrations of OTA by around 50% makes its use promising. This could lead to a decrease in concentrations to within permissible levels while also being a sustainable alternative.

#### 2.6.2. Significance of Variables

The statistical analysis has confirmed that two factors, pH and concentration, increase the efficiency of removing OTA; however, only the concentration has a significant effect. However, the factor of time showed that it needs to be further studied for optimization and maximization of adsorption, as high values of this factor may lead to a decrease in absorption, possibly due to the subsequent desorption of OTA from the bacterial membranes (see Figure 7, graphic Pareto chart).

The interaction between these variables also played a crucial role, as shown by the varying removal percentages across different runs. 

The derivatization of this equation, along with factors A, B, and C, results in the equation system. The experimental data were analyzed using the statistical software Statgraphics Centurion XVI. The polynomial model equations derived from the response surface analysis are presented in Equation (1).

(1) $$\begin{array}{ll} \%\;OTA\;removal & =46.4574+1.11111A-9.80972B+6.15417C+2.4123A^{2}\\ & -0.174074AB-0.975926AC-6.93009B^{2}-2.26019BC\\ & -5.25787C^{2} \end{array}$$

#### 2.6.3. Statistical Analysis Response, Regression Model, and Optimization of MAE

The model showed a strong fit with an *R*^2^ value of 95.26% and an adjusted *R*^2^ value of 86.73%. This indicates that the regression model can account for a significant amount of the variability in OTA removal efficiency. More details can be found in the supplementary information (see Table S4).

The standard error of the estimate was 3.693, suggesting that the model’s predictions closely match the actual values observed in the experiments.

The study used a Box–Behnken design (BBD) to optimize the process. Initially, 27 experiments were needed to explore three variables at three different levels. However, using the BBD, this number was efficiently reduced to 15. The data obtained from these experiments allowed for the creation of a statistical model to identify the optimal conditions for the removal process. The calculated values for the optimum removal are as follows: pH (−0.999; coded value) = 3.0, time (−0.829; coded value) = 75.39 min, and concentration (0.671; coded value) = 43.82 mg mL^−1^ for a 53.68% removal of OTA from the wine model solution.

## 3. Conclusions

Obtaining holocellulose from *Vitis vinifera* L. grape stems represents a significant advancement in the reuse of agro-industrial waste. It offers a sustainable and high-value-added alternative. The formation of a Holo-LP/Ch/Al polymeric complex, through the immobilization of *Lactobacillus plantarum* bacteria on holocellulose and its coating with chitosan and alginate, proved to be an effective strategy for stabilizing and protecting bacterial cells.

The Box–Behnken design was effective in optimizing the conditions for OTA removal using the polymeric Holo-LP/Ch/Al complex. The significant factors identified were pH, time, and concentration, with the best removal efficiency observed at a low pH (near 3.0). However, concentration was found to be the most critical factor in increasing OTA removal in the model solution. These findings can guide further optimization and application of polymeric complexes in the removal of OTA.

Considering the results, the incorporation of bacteria in the interstitial spaces of the Holocellulose and the subsequent coating of this complex with biopolymers make it a suitable system for bacterial immobilization and a potential alternative to reduce the concentration of mycotoxins from matrices as complex as wine.

## 4. Material and Methods

### 4.1. Stalk Grape Collection and Processing to Obtain Holocellulose

The stalks from the Cinsault wine grape were collected in the sector of Pachagua from Quirihue (−36.410723820608524, −72.56566704354464) in Ñuble region from Chile. The stalks were chopped, washed with distilled water for cleaning to remove grape residue, then dried in an oven at 80 °C for 24 h up to constant weight, then ground to powder at 36,000 rpm; the result (fine powder with a high surface area), was separated sieve through a 0.1 mm. Subsequently, 20 g of the dried powder was placed in a Soxhlet apparatus and treated with 250 mL of hexane (a non-polar solvent) at 90 °C for 6 h to remove lipophilic substances. The solvent was then removed in a vacuum evaporator at 40 °C.

The delignification process was based on previous studies with some modifications [77]. A solution of 5.0 g Sodium chlorite (NaClO_2_; 80% purity) with 10 g of the dried material was heated at 75 °C for 2 h with constant agitation. The mixture was allowed to cool to room temperature, and the fibers were then thoroughly washed with a water/acetone mixture (1:1). The phases were separated by filtration, and the solid was dried until a constant weight was obtained.

### 4.2. Formation of Holocellulose-LP/Chitosan Complex with Alginate Coating (Holo-LP/Ch/Al Complex)

The bacterium *Lactobacillus plantarum* 299v (LP) was used as a model probiotic. The LP strain was commercially obtained from BION, Merck, and stored frozen in de Man, Rogosa, and Sharpe (MRS) broth (Merck) with 15% (*v*/*v*) glycerol. To activate the LP for testing, it was incubated in MRS broth at 37 °C for 24 h in a 5% CO_2_ incubator and then a subculture in MRS agar. Wet cells were obtained by incubating the LP in 500 mL Erlenmeyer flasks containing 300 mL of MRS broth at 37 °C in anaerobic conditions. After 24 h of incubation, the wet cells were collected by centrifugation at 10,000 rpm at 4 °C for 10 min. The cell pellet was then collected and washed three times with 0.9% sterilized sodium chloride solution [41].

The dehydrated and bleached cellulosic materials were immersed in a bacterial mixture and water in a 1:1 ratio (*w*/*v*). They were then subjected to vacuum inclusion for 30 min at 60 °C to achieve complete bacterial inclusion, resulting in the formation of the Holo-LP complex.

The Holo-LP/Ch complex was prepared using the gel formation technique. First, 100 mg of Holo-LP complex was added to 10 mL of 1.0% (*w*/*v*) aqueous chitosan solution and stirred for 5 min to create an emulsion. This emulsion was then added to a 1.5% (*w*/*v*) sodium alginate solution. The resulting interaction between the anionic sodium alginate and cationic chitosan, along with the use of calcium chloride (1.5%) as a crosslinker, led to the formation of core–shell microcapsules, as shown in Figure 1. The microcapsules obtained were then washed with distilled water and freeze-dried at 4 °C. The resulting microparticles have a core made of the complex holocellulose–bacteria and a shell composed of chitosan and alginate crosslinked with calcium (Holo-LP/Ch/Al complex).

### 4.3. Thermogravimetric (TG) Characterization and Differential Thermogravimetric (DTG) Analyses of Complexes

All samples underwent thermogravimetric analysis using an STD 650 Thermal analyzer. Approximately 10 mg of each sample were placed onto a Pt crucible and then heated at a constant rate of 10 °C min^−1^ from 50 to 560 °C. Air was used as a reactive gas, with a mass flow of 50 mL min^−1^. Additionally, 50 mL min^−1^ of N_2_ was employed as a protective gas in the electronic balance.

Deconvolution of DTG curves using a mathematical deconvolution technique was performed to identify peaks. The objective was to conduct a comparative study to reduce polymeric components. This procedure is based on the functions of the peaks of DTG curves obtained due to the overlapping of signals [41,78].

### 4.4. Attenuated Total Reflection Fourier Transform Infra-Red (ATR-FTIR) Spectroscopy

The samples were analyzed with FTIR spectroscopy (Agilent Technologies, Santa Clara, CA, USA, Cary-360). The absorbance was measured from 500 to 4000 cm^−1^ with a resolution of 4 cm^−1^.

### 4.5. Color Determination (CIELab)

The cellulosic material and the holocellulose obtained were examined, and the color of the different samples was analyzed (five replicates) using a Nix Pro 2 Color Sensor (Nix Sensor Ltd., Hamilton, ON, Canada) and expressed in terms of the Hunter scale (L*, a*, b*). The schematic representation of the CIELab color system was obtained by a Nix Color change was expressed as ΔE* according to the following formula (see Equation (2)):

(2) $$\Delta E=((\Delta L^{*2})+(\Delta a^{*2})+{\left(\Delta b^{*2})\right)}^{1/2}$$

where ΔL*, Δa*, and Δb* represent the differences between the initial and final values [79], the determination of color change (see Table S1 in Supplementary Materials).

### 4.6. Scanning Electronic Microscopy (SEM)

Sample preparation for SEM involved placing all samples on carbon tape on a pin holder. A 30 nm layer of Au was then coated onto each sample using a 108 Auto Sputter Coater Cressington to prevent charging during observation. The morphology of the samples was studied using a Carl Zeiss EVO MA 10 scanning electron microscope at 20 kV.

### 4.7. Removal of Ochratoxin a by Holo-LP/Ch/Al Complex

In this study, we investigated the impact of different doses of Holo-LP/Ch/Al complex on the level of OTA in a wine model solution spiked with 2.5 mg L^−1^ of this mycotoxin. The wine model solution was prepared with a certain volume of Milli-Q water and 12% ethanol, adjusted to pH levels of 3.0, 3.5, and 4.0 using 0.1M acetic acid, and containing 5 g L^−1^ of L-tartaric acid.

The 2 mL mixture was contacted with different concentrations of the complex and then placed in a thermostatic shaking at 120 rpm for 60, 150, and 240 min, and concentrations of 10, 30, and 50 mg mL^−1^.

Subsequently, the samples were filtered using a 0.22 μm pore size disk of PDVF (Millipore Corporation, Billerica, MA, USA) to separate the complex. The resulting filtrate samples were then analyzed using high-performance liquid chromatography (HPLC). The capacity of each complex was determined using a specific Equation (3).

(3) $$\%\;OTA\;removal=\left(\frac{C_{0}-C_{f}}{C_{0}}\right)*100$$

where *C*_0_ is the initial concentration measured in the model solution, and *C*_f_ is the remaining concentration of OTA after the complex treatment.

#### 4.7.1. Analysis of OTA in Wine Model Solution by High-Performance Liquid Chromatography with Detector of Fluorescence (HPLC-FLD)

The amount of OTA was measured using an HPLC system coupled to a fluorescence detector (Agilent ChemStation, 1200, Santa Clara, CA, USA). The system was equipped with a low-pressure quaternary pump (Agilent 1200 model) and an autosampler (Agilent 1260 Infinity Autosampler). The column used was a LiChrospher RP-18 (250 mm length and 5 μm particle size) with a LiChrospher RP guard column select B (5 μm particle size). The analysis was conducted under the following conditions: a mobile phase consisting of H_2_O:ACN:CH_3_COOH (49.5:49.5:1, *v*/*v*), operated in isocratic mode at a flow rate of 1.2 mL min^−1^. The injection volume was 50 μL, and the analyte detection was performed at an excitation wavelength of 334 nm and emission wavelength of 460 nm. The run time was 7.0 min at room temperature, and the retention time (Tr) was 5.84 min. The analysis method was based on the description by Castro et al., 2022 [41], with some modifications.

#### 4.7.2. Limit of Detection (LOD), Limit of Quantitation (LOQ) and Linear Range

The calibration curve was developed using the OTA concentration range of 0.1–10 mg L^−1^. OTA was quantified using the limit of detection (LOD), which was calculated as 3 σ/S, where S represents the slope of the calibration curve, and σ is the standard deviation of the detector response. The limit of quantitation (LOQ) was determined as 10 σ/S. The analytical procedure was validated according to the Q2B methodology [80].

The value of σ was obtained from the standard deviations of the y-intercepts of the experimental calibration curves. The calculated LOD and LOQ were 0.054 mg L^−1^ and 0.016 mg L^−1^, respectively. The linear range for OTA determination was between 0.05 and 10.0 mg L^−1^.

### 4.8. Statistical Modelling Using Response Surface Methodology (RSM) and Box–Behnken Design (BBD)

The RSM technique was used to analyze and find the optimal design by fitting the second-order regression equation. RSM is an effective method to evaluate the effects of interactions of variables on responses. The experiments in RSM-BBD for optimizing were modeled and designed using Equation (4).

(4) $$N=2K\;\left(K-1\right)+C_{0}$$

where *N* is the number of experiments run, *K* is the number of variables (*K* = 3), and *C*_0_ is the number of center points. And the mathematical model with quadratic second-order regression equation was calculated using Equation (5) [81].

(5) $$Y=\beta_{0}+\sum_{i=1}^{k}\beta_{i}x_{i}+\sum_{i=1}^{k}\beta_{ii}x_{i}^{2}+\sum *\sum_{i<j}^{k}\beta_{ij}x_{i}x_{j}+\varepsilon$$

where *Y* is the response of removal of OTA in solution; *x_i_* and *x_j_* represent the independent variables pH, time, and concentration; *β*_0_ is the interception; *β_i_*, *β_i_*_i_, and *β_ij_* are the fitted equation of linear coefficients, quadratic coefficients and coefficients of an interaction effect, respectively, and to obtain a final estimation of the experimental error, three replications were performed, and X_1_, X_2_ and X_3_ were used in the experimental design matrix as pH, time (min.) and concentration (mg mL^−1^), respectively, and as a control method, the Box–Behnken model makes it possible to forecast the OTA removal performance in situations that were not evaluated by examining the variety of experimental conditions to offer a thorough evaluation of the behavior of the system, guaranteeing that the optimization process takes into consideration all conditions.

### 4.9. Statistical Analysis

The results were expressed as mean values ± standard deviation, and the data were analyzed using Statgraphic Centurion XVI, statistical software. The ANOVA test was used to compare the mean values between groups with a level of significance of *p* < 0.05.

## Figures and Tables

**Table 1:** The characteristic bands in FT-IR spectra of wood samples between 3390 and 895 cm^−1^.

Signal (cm^−1^)	Band Assignment	Ref.
3390–3367	O-H stretching in hydroxyl groups	[49]
3276	O_6_H_6_O_3_ intermolecular in cellulose *I*_β_ (streching)	[45]
2926	CH_2_ asymmetric stretch (in cellulose and lignin), CH stretch (in all three main components)	[45]
2980–2835	CH_2_, CH_2_OH in cellulose	[50,51]
1730–1726	C-O stretching in unconjugated ketone, carbonyl, and aliphatic groups (xylan)	[47]
1614–1602	Aromatic skeletal stretching (lignin)	[48]
1511–1509	Aromatic skeletal stretching (lignin)	[48]
1464	CH_3_ asymmetric stretch, CH_2_ scissoring in lignin and carbohydrates	[45]
1428–1427	CH_2_ deformation in lignin and carbohydrates	[46]
1374–1375	C-H deformation, CH_3_ symmetric deformation in carbohydrates	[52]
1330–1325	Phenolic OH; S ring plus G ring condensed (i.e., G ring substituted in pos. 5)	[53]
1317–1315	CH_2_ rocking vibration	[52]
1268	Guaiacyl ring and C-O stretch in lignin and xylan	[54]
1235–1230	Syringyl ring and C-O stretch in lignin and xylan	
1210	C-O-C stretching vibration in cellulose and hemicelluloses	[54]
1162	Asymmetric C-O-C in the ring (cellulose and hemicelluloses)	[55]
1058	C−O stretching mainly from C(3)−O(3)H in cellulose I	[46]
1034–1033	C−O and C−C stretching the ring in cellulose and hemicelluloses	[46]
1047–1040	C_alky_–O ether vibrations, methoxyl and b-O-4 C_alky_–O ether vibrations in guaiacol	[56]
896–895	C-groups, C_1_–H deformation in cellulose	[45]

**Table 2:** FTIR Bands of alginic acid and chitosan with assignments.

Signal (cm^−1^)	Band Assignment	Ref.
	Alginic acids	
3700–3000 (broad)	(broad) OH stretch	[68]
3000–2850	CH stretch	[72]
1722	C=O stretch of COOH	[73]
1596	antisymmetric COO-	[68]
1385, 1347	O-H deformation and C-O stretch modes	[68]
1237	skeletal vibration	[68]
1076	Stretch–COO–groups	[70]
	Chitosan	
3290–3180	O-H and N-H stretch	[69]
2914–2865	C-H stretch	[69]
1643	Amide I	[70]
1574	N-H bending from amine and amide II	[71]
1376	CH bending, CH_2_ wagging	[69]
1150	antisymmetric stretch C-O-C and C-N stretch	[68]

## Data Availability

The original contributions presented in this study are included in the article/Supplementary Materials. Further inquiries can be directed to the corresponding authors.

## Supplementary Materials

The following supporting information can be downloaded at: https://www.mdpi.com/article/10.3390/toxins17010026/s1. Table S1: Parameter of nonlinear curve fit (Gauss) from deconvolution of DTG spectra of control; Table S2: Parameter of nonlinear curve fit (Gauss) from deconvolution of DTG spectra of holocellulose; Table S3: Criteria to assess the color change (Castro et al. 2022b); Figure S1: Microscope image, blue arrow indicates *L. plantarum*. (A) Holocellulose; (B) Holo-LP complex; (C) Holo/Ch/Al; (D) Holo-LP/Ch/Al complex; Table S4: Experimental independent variables, levels, and results of percentage of removal of OTA; Table S5: Analysis of variance (ANOVA) for Absorption of OTA.
