# Supplementary material for: Holocellulose from a Winemaking By-Product to Develop a Biopolymeric System for Bacterial Immobilization: Adsorption of Ochratoxin A in Wine Model Solutions (Box–Behnken Design)
Source: Toxins (Basel). 2025 Jan 6;17(1):26. doi: 10.3390/toxins17010026 (PMC11768956; doi:10.3390/toxins17010026)
Supplement: Supplementary file 1 [file toxins-17-00026-s001.zip › toxins-3359179-supplementary.pdf]

toxins

MDPI

Supplementary material: Holocellulose from a Winemaking By-Product to Develop a Biopolymeric System for Bacterial Immobilization: Adsorption of Ochratoxin A in Wine Model Solutions (Box–Behnken Design)

Verónica Carrasco-Sánchez, V. Felipe Laurie, Marcelo Muñoz-Vera and Ricardo Ignacio Castro

Table S1. Parameter of Nonlinear Curve Fit (Gauss) from deconvolution of DTG spectra of control.

Model	Gauss		
**Equation**	**y=y0 + (A/(w*sqrt(pi/2)))*exp(-2*((x-xc)/w)^2)**		
Plot	Peak1(C)	Peak2(C)	Peak3(C)
y0	-0.01015 ± 8.7408E-4	-0.01015 ± 8.7408E-4	-0.01015 ± 8.7408E-4
Xc	229.90341 ± 0.31454	308.36949 ± 0.08221	414.2442 ± 0.88859
W	73.78002 ± 0.74651	54.69204 ± 0.17416	136.99933 ± 2.71526
A	-15.57867 ± 0.21524	-32.79359 ± 0.20942	-11.82771 ± 0.35888
Reduced Chi-Sqr		1.76751E-4	
R-Square (COD)		0.99105	
Adj. R-Square		0.99104	

Table S2. parameter of Nonlinear Curve Fit (Gauss) from deconvolution of DTG spectra of Holocellulose.

Model	Gauss		
**Equation**	**y=y0 + (A/(w*sqrt(pi/2)))*exp(-2*((x-xc)/w)^2)**		
Plot	Peak1(C)	Peak2(C)	Peak3(C)
y0	-0.02611 ± 3.74851E-4	-0.02611 ± 3.74851E-4	-0.02611 ± 3.74851E-4
xc	263.06522 ± 0.48635	317.89939 ± 0.20157	426.18845 ± 1.03834
w	58.51097 ± 0.53551	51.54263 ± 0.23927	110.69399 ± 2.60128
A	-18.0544 ± 0.32555	-29.47095 ± 0.33518	-4.65206 ± 0.13741
Reduced Chi-Sqr		1.48905E-4	
R-Square (COD)		0.99412	
Adj. R-Square		0.99412	

Table S3. Criteria to assess the color change (Castro et al. 2022b).

Parameter	Description	Sample		
		**L***	**a***	**b***
ΔE < 0.2	Invisibles changes			
0.2 < ΔE < 2	Small changes			
2 < ΔE < 3	Color changes visible by high quality filter			
3 < ΔE < 6	Color changes visible medium quality filter			
6 < ΔE < 12	Distinct color changes			
ΔE > 12	A different color			

Control	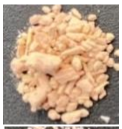	60.5 ± 2.3	6.0 ± 0.1	20.8 ± 2.1
Holocellulose	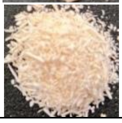	76.1 ± 5.1	3.6 ± 0.8	16.0 ± 0.5

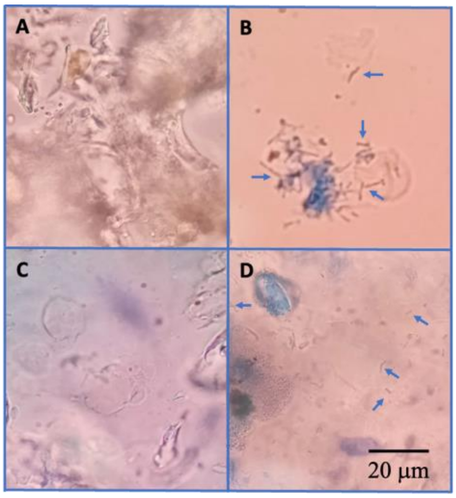

Figure S1. Microscope image, blue arrow indicates *L. plantarum*. (**A**) Holocellulose; (**B**) Holo-LP complex; (**C**) Holo/Ch/Al; (**D**) Holo-LP/Ch/Al complex.

Table S4. Experimental independent variables, levels and results of percentage of removal of OTA.

	**Coded Value**			**Uncoded Value**			Results
**Samples**	**pH**	**Time (min.)**	**Conc.**	**pH**	Time (min.)	Conc. (mg mL^{-1})	% removal OTA
1	0.0	0.0	0.0	3.5	150	30	47,98333
2	-1.0	-1.0	0.0	3.0	240	30	48,89814
3	1.0	-1.0	0.0	4.0	60	30	54,43888
4	-1.0	1.0	0.0	3.0	240	30	25,44629
5	1.0	1.0	0.0	4.0	240	30	30,29074
6	-1.0	0.0	-1.0	3.0	150	10	33,79444
7	1.0	0.0	-1.0	4.0	150	10	34,99814
-8	0.0	0.0	0.0	3.5	150	30	47,39444
9	-1.0	0.0	1.0	3.0	150	50	49,83518
10	1.0	0.0	1.0	4.0	150	50	47,13518
11	0.0	-1.0	-1.0	3.5	60	10	34,46481
12	0.0	1.0	-1.0	3.5	240	10	23,54629
13	0.0	-1.0	1.0	3.5	60	50	49,5129
14	0.0	1.0	1.0	3.5	240	50	29,5537
15	0.0	0.0	0.0	3.5	150	30	43,9944

Table S5. Analysis of variance (ANOVA) for Absorption of OTA.

Source	Sum of squares	DF	Mean of square	F-value	p-value
A:Factor_A	9.87655	1	9.87655	0.72	0.4337
B:Factor_B	769.844	1	769.844	56.43	**0.0007**
C:Factor_C	302.989	1	302.989	22.21	**0.0053**
AA	0.214838	1	0.214838	0.02	0.9050
AB	0.121205	1	0.121205	0.01	0.9286
AC	3.80972	1	3.80972	0.28	0.6198
BB	177.328	1	177.328	13.00	**0.0155**
BC	20.4335	1	20.4335	1.50	0.2755
CC	102,075	1	102,075	7,48	0,0410
Error total	68,2142	5	13,6428		
Total (corr.)	1440,15	14			

R^2 = 95.2634.
R^2 adjusted per d.f. = 86.7375.
Error standard. = 3.69362.
Error absolute medio = 1.83927.
